# Chemosensory function of *Varroa* gnathosoma: transcriptomic and proteomic analyses

**DOI:** 10.1007/s10493-024-00952-1

**Published:** 2024-10-23

**Authors:** Beatrice T. Nganso, Nurit Eliash, Kannan Mani, Noa Sela, Alejandro Villar-Briones, Angelina Fathia Osabutey, Ada Rafaeli, Alexander S. Mikheyev, Victoria Soroker

**Affiliations:** 1 Department of Entomology, Institute of Plant Protection, Agricultural Research Organization, the Volcani Center, Rishon LeZion, Israel; 2 International Centre of Insect Physiology and Ecology (icipe), Nairobi, Kenya; 3 Ecology and Evolution Unit, Okinawa Institute of Science and Technology Onna-son, Okinawa, Japan; 4 Shamir Research Institute, Rishon LeTsiyon, Israel; 5 University of Haifa, Haifa, Israel; 6 Bioinformatics Unit, ARO Volcani Center, 68 HaMaccabim Road, P.O.B 15159, Rishon LeZion, 7528809 Israel; 7 Instrumental Analysis Section, Okinawa Institute of Science and Technology Graduate University, Okinawa, Japan; 8 Institute of Postharvest and Food Sciences, Agricultural Research Organization, the Volcani Centre, Rishon Lezion, Israel; 9 Research School of Biology, Australian National University, Canberra, ACTRR Australia

**Keywords:** Sensory organs, Lipid carrier proteins, Membrane-bound receptors and proteins, Honey bee parasitic mite

## Abstract

In this study, we evaluated the role of the gnathosoma (mouthparts) in chemosensing of the most devastating honey bee parasite, *Varroa destructor* mite. Through transcriptomic analysis, we compared the expression of putative chemosensory genes between the body parts containing the main chemosensory organs (the forelegs), gnathosoma and the rest of the body devoid of these two body parts. Furthermore, we checked the presence of chemosensory-related transcripts in the proteome of the gnathosoma. Our comparative transcriptomic analysis revealed the presence of 83 transcripts with known characteristic conserved domains belonging to eight chemosensory gene families in the three *Varroa* transcriptomes. Among these transcripts, 11 were significantly upregulated in the mite’s forelegs, compared to 8 and 10 in the gnathosoma and body devoid of both organs, respectively. Whilst the gnathosoma and the forelegs share similar expression of some putative lipid carrier proteins, membrane-bound receptors, and associated proteins, they also differ in the expression profiles of some transcripts belonging to these protein families. This suggests two functional chemosensory organs that may differ in their chemosensory function according to specific characteristics of compounds they detect. Moreover, the higher expression of some chemosensory transcripts in the body devoid of forelegs and gnathosoma compared to the gnathosoma alone, may suggest the presence of additional function of these transcripts or alternatively presence of additional external or internal chemosensory organs. Insights into the functional annotation of a highly expressed gustatory receptor present in both organs using RNA interference (RNAi) are also revealed.

**Supplementary Information:**

The online version contains supplementary material available at 10.1007/s10493-024-00952-1.

## Introduction

Olfaction, as well as gustation, are essential for animal survival and fitness, allowing behavioral modulation according to environmental input thus optimizing the detection of food, mates, and enemy-avoidance *via* volatile and contact chemicals. However, the molecular structure and function of the chemosensing organs are not well known in non-insect arthropods in general, and Acari (mites and ticks) in particular. As a model organism, we are studying the obligatory ecto-parasitic mite, *Varroa destructor* (Anderson and Trueman) (Parasitiformes, Mesostigmata, Varroidae), considered as the major pest of the Western honey bee almost worldwide (Traynor et al. 2020). The life cycle of the *Varroa* mite is tightly synchronized with the development of the honey bee and the health status of the colony. It consists of two main phases: a reproductive and a non-reproductive (i.e., dispersal) phase (Martin 2001; Rosenkranz et al. 2010). During the dispersal phase, the female mite usually parasitizes an adult bee either nurse or forager and spreads within or between the colonies. During the reproductive phase, the female mite parasitizes the cell containing a fifth instar honey bee larva and reproduces within the cell till the emergence of the adult bee (Martin 2001). Detection of the honey bee stage and colony condition occurs via the mite’s eavesdropping on host honey bee chemical cues (Frey et al. 2013; Nazzi and Le Conte 2016; Pernal et al. 2005; Plettner et al. 2017). Information on *Varroa*’s host searching and selection behavior and the nature of chemical cues from the host bee and their environment that regulate its reproduction suggests that both high (e.g. geraniol, 8-heptadecene, nerolic acid) and low (fatty acid methyl and ethyl esters, hydrocarbons C21-C29) volatile molecules are perceived by the mite (Nazzi and Le Conte 2016; Plettner et al. 2017; Soroker et al. 2019).

The occurrence of a few chemosensory organs might be adaptive to ensure the survival of most if not all studied terrestrial arthropods. It enables not only to create a broader chemical picture by receiving a wide range of environmental cues but also a backup, to maintain chemosensory abilities in case of a failure in one of the organs. In the case of the honey bee parasitic *Varroa* mite, two sites with putative chemosensory sensilla are the forelegs and the gnathosoma (Dillier et al. 2006). The fact that while walking *Varroa* holds their forelegs above the ground in a similar manner to antennae in other arthropods points towards the role of the forelegs in volatile detection. The presence of nine chemosensory sensilla located inside a pit and 9 more sensilla surrounding it on the distal dorsal part of each foreleg, analogous to Haller’s organ found in ticks, further indicated foreleg function in *Varroa* host chemosensing (Dillier et al. 2006). Liu and Peng (1990) reported that some of the *Varroa* pedipalpi setae are tip pore sensilla and thus could play a potential role in its host chemosensing, but (Dillier et al. 2006), did not indicate any chemosensory sensilla in the pedipalpi and stated that their four segments are covered by strong trichoid setae and in addition, each distal segment of the pedipalpi contains long setae.

Focusing on the role of host sensing by *Varroa* forelegs, we showed their clear ability to sense both nurse and forager honey bee volatiles as well as the honey bee pheromone, E-β-ocimene (Eliash et al. 2014; Singh et al. 2015). Moreover, we showed that disruption of foreleg volatile chemosensing by specific dialcoxybenzenes, cyclopentenol ethers and N, N-Diethyl-m-toulamide (DEET) is reflected in changes in host preference and detection abilities by free moving mites (Eliash et al. 2014; Singh et al. 2015). On the other hand, our recent study demonstrated that host recognition ability in foreleg varnished mites dropped to about 40% within the first hour of the bioassay relative to about 80% of foreleg-unvarnished mites (Nganso et al. 2020). Additionally, only about 20% of mites remained on the host three hours following mechanical blocking of the main olfactory organ on the forelegs and only one mite was feeding relative to about 80% and 35% of foreleg-unvarnished mites, who remained and fed on the host, respectively. Overall, these findings suggested that the sensilla of the gnathosoma have relatively minor chemosensory function when compared to those of the forelegs.

Focusing on the forelegs as the main chemosensory organ and based on assumption of general similarity in the chemosensory mechanisms in arthropods, we and other colleagues analyzed transcriptome of *Varroa* forelegs. We have not found chemosensory proteins (CSPs) and odorant receptors (ORs) but identified some chemosensory-related gene transcripts. These belong to eight protein families, some are conserved across Arthropoda but others are arachnid-specific (Eliash et al. 2017, 2019; Iovinella et al. 2018; Zhu et al. 2021a, b). These include transcripts of soluble odorant carrier proteins as well membrane associated proteins and receptors. Among carrier proteins, transcripts of the members of three groups were identified: Niemann-Pick type C2 (NPC2) protein, odorant binding proteins (OBPs) and lipocalins, while among membrane-bound receptors and associated proteins, members of six groups were detected: ionotropic glutamate receptors (IGRs), ionotropic receptors (IRs), gustatory receptors (GRs), sensory neuron membrane proteins (SNMPs), transient receptor potential proteins (TRPs) and epithelial Na + channel (ENaCs) proteins. Among these chemosensory gene families, some were found to be significantly up-regulated in the forelegs compared to the other legs; thereby implicating their putative role in *Varroa* host chemosensing (Eliash et al. 2019). On the other hand, proteomic analyses of *Varroa* forelegs only detected proteins belonging to the OBP, NPC2, IGR and SNMP families (Eliash et al. 2019; Iovinella et al. 2018), with few OBP and NPC2 proteins significantly upregulated in this chemosensory organ (Eliash et al. 2019).

Recently, we demonstrated via RNA interference that silencing two NPC2 transcripts evoke different physiological effects on the mites. Silencing one highly expressed and foreleg-specific NPC2 transcript (*LOC111247660*), referred to as *Vd40090* in (Eliash et al. 2017), reduced feeding and reproduction of the mite without affecting host reaching ability, suggesting its involvement in the detection of short-range host cues (Nganso et al. 2021). In contrast, silencing the other NPC2 transcript (*LOC111247342*) referred to as *Vd74517* in (Eliash et al. 2017) significantly disrupted only the host reaching ability, thus indicating the crucial role of this putative odorant carrier protein in the detection of long-range host cues (Mani et al. 2022). Additionally, recently via an exclusion of the chemosensory appendages (forelegs, gnathosoma or both), we showed that in the absence of the forelegs, the expression level of at least one of the putative odorant binding proteins belonging to the NPC2 family previously reported in the forelegs (Eliash et al. 2019) are expressed in the gnathosoma (Nganso et al. 2021).

Whilst we know the identity of some putative chemosensory-related transcripts in *Varroa*’s forelegs (Eliash et al. 2019, 2017; Iovinella et al. 2018), and the role of a few of them in *Varroa*-host interaction (Nganso et al. 2021); Mani et al. 2022), the identity of those associated with gnathosoma and their functional significance are not well understood. So far, it is only the presence of lipid carrier proteins belonging to the OBP and NPC2 families that was reported in *Varroa* mouthparts via proteomic analysis (Iovinella et al. 2018). Therefore, our aim in this study was to compare the expression profile of putative chemosensory genes between the two chemosensory organs and to functionally annotate one highly expressed gustatory receptor present in both organs using RNA interference (RNAi).

## Materials and methods

### Collection of mites

Experimental honey bee colonies of *Apis mellifera ligustica* maintained in Langstroth hives at the Agricultural Research Organization, Volcani Center, Israel were used in the course of this study. These experimental colonies were not treated against *V. destructor* and received 60% sugar solution and 70% pollen cake seasonally when needed. The mites were collected from honey bees in these colonies by the sugar shake method (Dietemann et al. 2013). The mites were kept on moist filter paper and dissected within an hour following collection.

### Transcriptomics

#### Total RNA extraction

Within an hour of mite collection, an individual mite was put immediately into a 1.5 ml Eppendorf tube dipped in liquid nitrogen or was first dissected using a fine surgical blade (size no. 11) under a stereo microscope (Olympus SZX12, Shinjuku-ku, Tokyo, Japan). Three groups of samples were collected: (1) gnathosoma only, (2) mites without both the gnathosoma and forelegs (WB_minus), and (3) mites with both the gnathosoma and forelegs (WB_plus). Each treatment group contained a pool of approximately 300 individuals in four replicates (in total 1200 mites per treatment group). The samples were subsequently kept at – 80 ºC until total RNA extraction. Total RNA was extracted from each replicate as described before by Nganso et al. (2021). In short, samples in the 1.5 ml Eppendorf tubes were dipped in liquid nitrogen, ground and extracted for total RNA using a Geneall Kit (Geneall, Seoul, South Korea) following the manufacturer’s protocol. Elimination of genomic DNA contamination from the RNA samples was done using the TURBO DNA-free™ Kit (Thermo Fisher Scientific, Lithuania, USA) according to the manufacturer’s instructions. The samples were eluted from the column with 25 µl of RNase-free water supplied within the GeneAll kit before checking the quality and quantity of the RNA samples on a NanoDrop 2000 spectrophotometer (Thermo Scientific, Wilmington, DE, USA). The RNA samples were subsequently kept at – 80 ºC until shipment in dry ice to the Okinawa Institute of Science and Technology (OIST) in Japan for transcriptomic analysis.

#### RNA library preparation and sequencing

RNA quality and quantity were measured by NanoDrop spectrophotometer (Thermo Fisher Scientific, Japan), and Bioanalyzer 2100 with RNA Pico kits (Agilent, Japan). Libraries were prepared using Nextera XT Prep kit (Illumina, Japan) as described before (Aird et al. 2013). In brief, 200 ng of total RNA were used to prepare cDNA libraries, amplified in 16 PCR cycles, and purified using 16% PEG SPRI. The cDNA concentration was measured using Qubit fluorometer (Thermo Fisher Scientific, Japan), and quality checked using 4200 TapeStation with a high-sensitivity D5000 kit (Agilent, Japan). For sequencing, 0.2 ng of amplified cDNA were used in two lanes of Illumina HiSeq 2500 at the OIST sequencing center in 250 paired-end mode, according to manufacturer’s specifications.

#### Mapping, transcriptome analysis and chemosensory-related gene annotations

The raw read sequences from the three RNA samples were uploaded to the National Centre for Biotechnology Information (BioProject accession PRJNA872955). The reads were cleaned to remove adaptor sequences and reads with low quality scores using Trimmomatic software (Bolger et al. 2014). Cleaned reads were mapped to the reference genome of *Varroa* mite (GCF_002443255.1) (Techer et al. 2019) using Tophat 2 with the default parameters (Kim et al. 2013). Additionally, the former clean reads extracted and sequenced previously from the forelegs and rear legs of *Varroa* mite generated by (Eliash et al. 2019) were also mapped to the reference genome of the mite. Quantification and de-novo analysis of the new transcript was done using cufflinks, cuffquant and cuffnorm software package suit (Trapnell et al. 2012). Subsequently, the principal component analysis (PCA) was calculated among the three groups: gnathosoma, WB_minus and WB_plus using the function prcomp in R. Counts were normalized and P-values and log2fold change (the rate of change between the two values after logarithmic transformation) were computed by the DESeq package (Anders et al. 2010). Based on the multiple hypothesis correction (Benjamini and Hochberg 1995), P-values were adjusted (padj). The threshold for significant differential gene expression was set at padj ≤ 0.05 and log2 fold change ≥ 1. The Venn diagram was also constructed using the tool Venny version 2.1.0 (Oliveros 2007–2015) to show the distribution of differentially expressed genes among the three RNA groups. Following transcriptomic analysis, a protein database was constructed from the transcriptome assembly of the gnathosoma only to be used for proteomics analysis. Each oriented and un-oriented transcript was translated into amino acids using Transdecoder software (Haas, n.d.). In order to search for orthologues of all the assembled contigs belonging to OBP, NPC2, CSPs, ORs, IGRs, IRs, GRs, SNMPs, TRPs and ENaCs chemosensory-related families previously searched in *Varroa*’s forelegs and rear legs (Eliash et al. 2017, 2019), BLASTx was used along InterProScan (Quevillon et al. 2005) to identify the conserved domains specific to each transcript group as described before (Eliash et al. 2017, 2019). We also searched for transcripts of the nine lipocalins found in the genome of *V. destructor* (XP_022658018, XP_022661284, XP_022665322, XP_022671613, XP_022662731, XP_022653636, XP_022653633, XP_022664566, XP_022663986) (Zhu et al. 2021b) in the transcriptomes of the three RNA groups of this study and those of *Varroa*’s forelegs and rear legs (Eliash et al. 2019).

### Proteomics

#### Protein extraction

All chemicals were purchased from Sigma Aldrich (St Louis MO, USA). Proteins were extracted from *Varroa* gnathosoma samples only (a pool of at least 300 individuals, in four replicates as described above). Samples were manually crushed after adding 400 µl of SDT lysis buffer (0.1 M Tris pH 7.6, 0.1 M dithiothreitol, 4% sodium dodecyl sulfate) and placed in 4 °C overnight before subjecting to heating at 95 °C for 10 min followed by cooling for 5 min. at room temperature. Protein quantification in each sample with Bradford method (Bio-Rad Protein Assay) was done before shipping the samples in dry ice to OIST in Japan for proteomic analysis.

#### Protein digestion and purification

We used the S-Trap method for protein preparation. First, the crude protein extracts were sonicated (QSONICA Q800R, Thermo Fisher Scientific), heated at 60 ºC for 30 min and alkylated by incubating in iodoacetamide (Wako, Japan) for 30 min in darkness. After centrifugation, the supernatant was used for S-Trap digestion following the procedure described by (HaileMariam et al. 2018). In brief, protein concentration was measured using MilliQ Direct Detect IR spectrometer (Merck, Japan), and 50 µg of each sample were acidified with phosphoric acid (Wako, Japan). After adding triethylammonium bicarbonate (Sigma, Japan) in methanol buffer (Thermo Optima, Japan), the samples were passed through a S-Trap tip, and digested with Trypsin (Promega, USA) at 37 ºC overnight. The samples were then washed with 0.1% formic acid in water solution (Thermo, Japan), followed by 0.1% formic acid in 60% acetonitrile (Wako, Japan), vacuum dried, resuspend with 1% acetic acid, 0.5% formic acid and sonicated for 5 min. The proteins were then purified following the StageTip protocol (Rappsilber et al. 2007), vacuum dried and resuspended in 1% acetic acid, 0.5% formic acid prior to liquid chromatography and mass spectrometry (LC/MS) analysis.

#### Liquid chromatography and mass spectrometry

LC/MS was carried out using an ACQUITY M-Class UPLC (Waters corporations) connected to Orbitrap Fusion Lumos mass spectrometer (Thermo Fisher). Five µl of sample were loaded in 0.1% formic acid in acetonitrile (solvent B) onto a trap column (nanoACQUITY UPLC 2G-V/M Trap 5 μm Symmetry C18, 180 μm x 20 mm, Waters). Peptides were separated on the analytical column (nanoACQUITY UPLC HSS T3 1.8 μm, 75 μm x 150 mm, Waters) at 40 ºC at a flow rate of 500 nanoLiter/min using a gradient starting from 10% B (initial conditions) followed by 10% B (0–8 min), 95% B (8–8.3 min) and 10% B (8.3–14 min). The mass spectrometer was operated in positive ion mode at a spray voltage of 2.4 kV, in duration of 120 min, resolution of 120,000, scan range of 400-1,500 m/z, AGC target of 4e5 with a maximum injection time of 50 msec. The internal mass calibration was 445.12003 m/z, and the time between master scans lasted for 3 s. We applied the following filters: include charge states to 2–7, dynamic exclusion after 1 time, 12 s, exclude isotopes and the intensity set to higher than 5.0e4. in the data dependent scan, we set the isolation window to 1.2 m/z, collision energy mode was fixed, HCD Collision Energy was 25%, detector resolution set to 15,000, maximum injection time set to 40 msec, and the data type is Centroid.

#### Proteomic data processing and analysis

Proteome Discoverer software (Thermo Fisher scientific, version 2.2), was used to search against combined databases including: cRAP (for contaminants), predicted proteins in gnathosoma based on the current transcriptome study and predicted proteins based on *Varroa* whole genome sequencing (https://www.ncbi.nlm.nih.gov/protein/?term=varroa+destructor*).* The minimum and maximum precursor masses were set to 350 and 5,000 Da respectively, total intensity threshold set to 100, and the minimum pick count as 5. A maximum of two trypsin mis-cleavages were allowed with a precursor mass tolerance of 20 ppm, fragment mass tolerance of 0.7 Da, maximum peptide length of 144 and minimum of 7 amino acids. Carbamidomethyl + 57.021 Da (C) was selected as static modification, and deamidated + 0.984 Da (N, Q) and oxidation + 15.994915 (M, P) as dynamic modifications. We set the false discovery rate (FDR) to 0.01 for protein and peptide levels, and the data was normalized to the total protein abundance. The mass spectrometry proteomics data have been deposited to the ProteomeXchange Consortium (http://proteomecentral.proteomexchange.org) via the PRIDE partner repository (Perez-Riverol et al. 2022), with the dataset identifier PXD053065.

### Silencing of target GR transcript (LOC111245174)

We designed two sets of primers from two different sources for silencing the target GR transcript (Supplementary Table S1). To determine the amplification efficiency of these pairs of primers, the total RNA was extracted from a pool of five mites in eight biological replicates using the GeneAll Kit (Seoul, South Korea) following the manufacturer’s protocol. The concentration and quality of each RNA sample was then checked using a NanoDrop 2000 spectrophotometer (Thermo Scientific, Wilmington, DE, USA). Single-strand cDNA (20 ng/µL) was generated with the RNA samples (0.4 µg) using the qPCRBIO cDNA Synthesis Kit (Thermo Scientific, Waltham, MA, USA) following the manufacturer’s instructions. Subsequently, these cDNA samples were used to verify the amplification efficiencies of each set of primers using both PCR and RT-qPCR as in Nganso et al. (2021). Following successful amplification, the silencing primers with T7 promoters were purchased and used for *LOC111245174*-dsRNA syntheses as described in detail by Nganso et al. (2021).

The silencing of the target transcript was performed by a non-invasive method of dsRNA delivery to *Varroa* mites as in Nganso et al. (2021). A total of 60 adult mites were soaked in 50 µL of *LOC111245174*- dsRNA (2.5–4 µg/µl) in 10% NaCl (saline) solution in five biological replicates of 12 mites each. The control mites were soaked in 10% saline solution in eight biological replicates of 12 mites each. To test for silencing of *LOC111245174* transcript, total RNA was extracted from pools of mites that had recovered 15 h after soaking as described above. The cDNA synthesized from the RNA sampled were used for RT-qPCR using a second set of primers that did not overlap with the pairs of primers used for *LOC111245174*-dsRNA syntheses (Supplementary Table S1). The relative expression level of target transcript in each treatment category was calculated after normalization with 18 S rRNA using the 2^−ΔΔCT^ approach.

### Statistical analysis of proteomic data

For the proteomics analysis, the results from Proteome Discoverer were imported to Perseus software version 1.6.14.0 (https://maxquant.net/perseus/*)*, and the abundance data was filtered for contaminants and missing values. Log2 transformed data was used to produce a volcano plot using a two-sided t-test, with 250 randomizations, FDR = 0.05 and s0 = 1. A one-way ANOVA followed by a post hoc Tukey–Kramer test was used to compare the expression levels of the LOC111245174 transcripts among the three treatment groups (mites soaked in saline, 2.5–4 µg/µl LOC111245174-dsRNA) in JMP^®^, 14, SAS Institute Inc., Cary, NC, 1989–2019.

## Results

### Transcriptomics and differential gene expression analysis

**Fig. 1:**
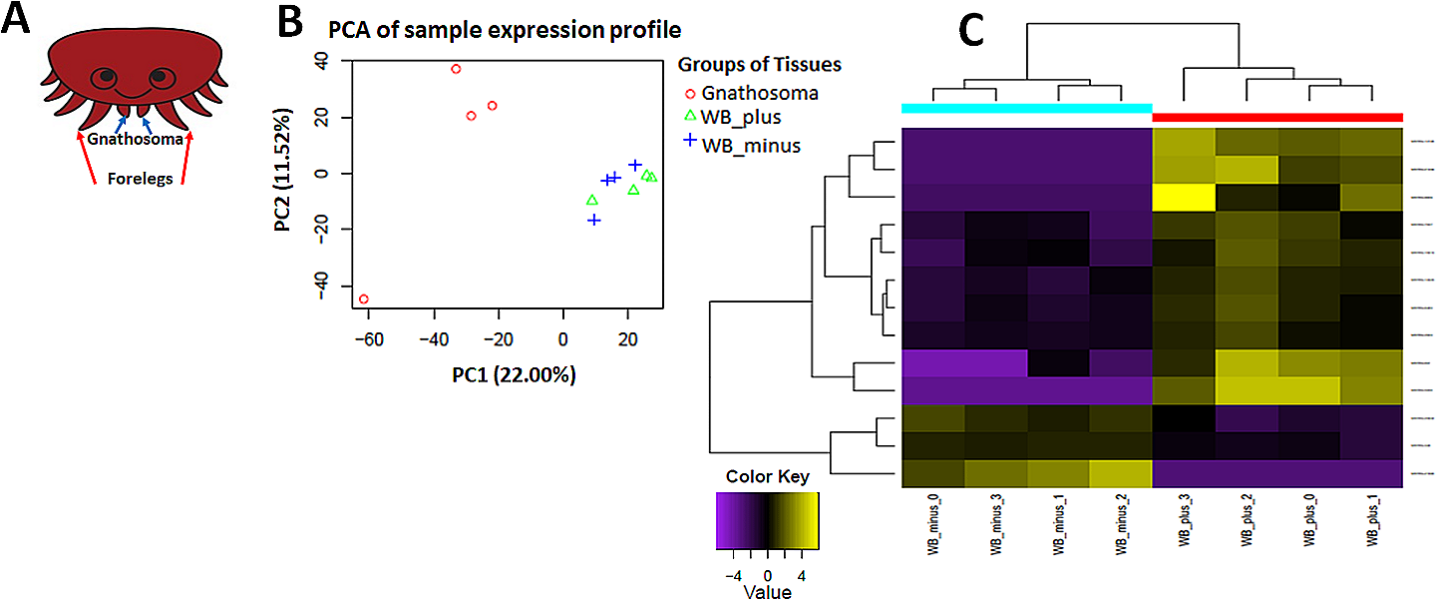
Transcriptome of differential gene expression for the three RNAs groups: gnathosoma only (gnathosoma), mites without gnathosoma and forelegs (WB_minus), and mites with both the gnathosoma and forelegs (WB_plus). (**A**) A schematic diagram of a *Varroa* mite, dorsal view: marked with red arrows is the pair of forelegs and marked with blue are the gnathosoma. (**B**) Principal components analysis (PCA) of gene expression separated the samples by their groups. The values used for the analysis are based on the 2067 differentially expressed genes with threshold of FDR < 0.05 and log2FC greater than one or lower than minus one. (**C**) Heat map of the 13 differentially expressed genes between the WB_minus and WB_plus. Log2 of the normalized reads are displayed as the color scale with downregulated genes in purple and upregulated genes in yellow

**Fig. 2:**
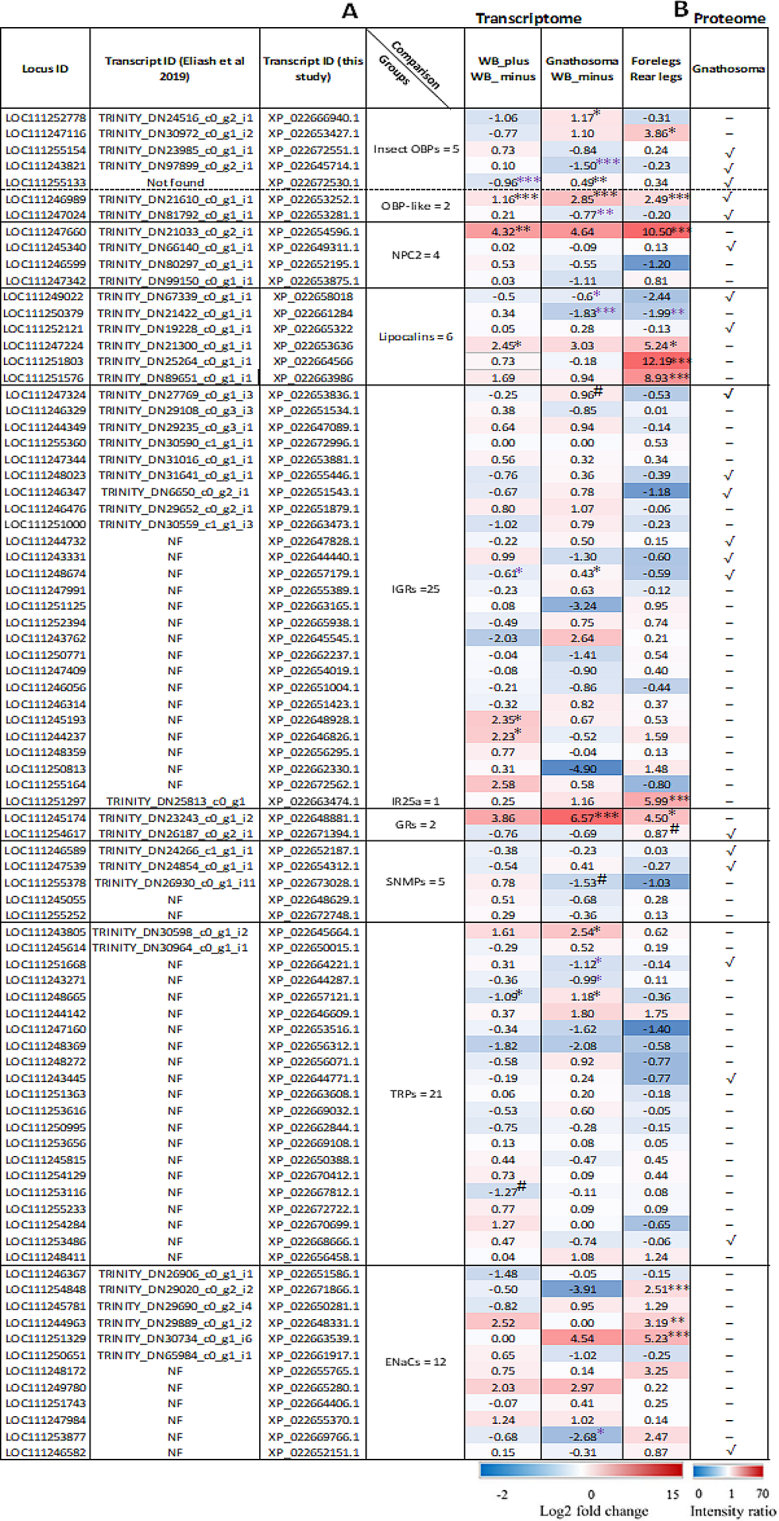
(**A**) Heat map of the chemosensory related differentially expressed gene transcripts. 83 transcripts identified in eight chemosensory groups, in three RNAs comparisons: gnathosoma, mites without gnathosoma and forelegs (WB_minus), and mites with both the gnathosoma and forelegs (WB_plus). Log2 fold change of the normalized reads are displayed at the bottom of the heat map, in colour scale (highest value = 15, lowest value = -2). Black asterisks “*”, “**”, “***” indicate significantly upregulated genes in the different comparisons (log2 fold change ≥ 1; Padj ≤ 0.05, 0.001, 0.0001); “#” indicates those that were close to significance (log2 fold change ≥ 1; Padj =  0.05; and “NF” indicate new transcripts detected in this study that was not previously reported by (Eliash et al. 2019) in the transcriptomes of the forelegs and rear legs of Varroa. Purple asterisks “*”, “**”, “***” indicate significantly downregulated genes in the different comparisons (log2 fold change ≤ -1; Padj ≤ 0.05, 0.001, 0.0001). (**B**) Twenty-one proteins identified in the proteome of the gnathosoma. Average intensity ratios are displayed at the bottom of the heat map, in colour scale (highest value = 70, lowest value = 0). OBPs (odorant-binding proteins); NPC2 (Niemann-Pick type C2 protein); IGRs (ionotropic glutamate receptors); IRs (ionotropic receptors); GRs (gustatory receptors); SNMPs (sensory neuron membrane proteins); TRPs (transient receptor potential proteins); ENaCs (degenerin/epithelial Na+ channel proteins)

Twelve RNAseq libraries of three types of *Varroa* tissues (with four replicates each): gnathosoma (Fig. 1A), whole body without gnathosoma (WB_minus), and whole body (WB_plus), were mapped to *V. destructor* genome (Techer et al. 2019). Principal component analysis was calculated based on a normalized count table. The PCA shows that the gene expression profiles of gnathosoma clearly differs from the other two groups: the WB_minus and the WB_plus (Fig. 1B and Supplementary Fig. S1). Of the 2,067 differentially expressed genes, 1,855 (89.74%) differ between WB_plus and gnathosoma and 961 (46.49%) differ between WB_minus and gnathosoma. In contrast, the gene expression profile of the WB_minus and WB_plus groups seems to be highly similar (Fig. 1B and Supplementary Fig. S1). In fact, only 13 (0.63%) of the 2,067 differentially expressed genes differ between both groups of tissues (Fig. 1C and Supplementary Table S2). Among these 13 genes, one (LOC111246989) was a chemosensory transcript belonging to the OBP-like group (Supplementary Table S2). It was found to be highly expressed in the WB_plus compared to the WB_minus and in both forelegs and gnathosoma and was even detected in gnathosoma proteome (Fig. 2 ).

### Annotation and differential gene expression analysis of chemosensory-related transcripts and proteins

In the current study, we found 83 transcripts belonging to OBP, NPC2, lipocalins, IGR, IR, GR, SNMP, TRP and ENaCs chemosensory-related families in the three transcriptome libraries studied herein: gnathosoma, WB_plus and WB_minus (Table 1, and Fig. 2). No odorant receptor or chemosensory protein were found. The re-mapping of *Varroa* foreleg and rear legs transcriptomes generated by Eliash et al. (2019) to the reference genome of the mite also revealed the presence of all these 83 transcripts (Table 1; Fig. 2). These transcripts were classified into their respective families based on the presence of their conserved characteristic domains and GO terms (see Table 1 in (Eliash et al. 2017) and Table 2 in (Eliash et al. 2019). The identified chemosensory related transcripts included both lipid carrier proteins (17 transcripts), presumably carrying and solubilizing odors, and membrane-bound proteins, hypothetically acting as chemoreceptors or co-receptors (66 transcripts). Of the carrier proteins, seven belong to the OBP (five were insect OBP transcripts and two were OBP-like transcripts), four to the NPC2 families, and six to the Lipocalin family. Among the membrane-bound proteins, we identified 26 ionotropic receptors (25 of the IGR family and one from the IR subfamily), two GR proteins, five SNMPs, 21 TRPs and 12 ENaCs transcripts (Table 1). We further checked if these transcripts are also translated into proteins in the *Varroa* gnathosoma. The *Varroa* gnathosoma proteome yielded a total of 2,795 proteins, of which 1,758 were supported by more than one peptide (Supplementary Table S3). Of the chemosensory transcripts, eight out of the 17 lipid carrier proteins were also identified in the proteome, and 13 out of the 66 membrane-bound proteins had a corresponding protein (Fig. 2 ).

**Table 1:** Transcripts of putative chemosensory genes upregulated in mite’s body with both the gnathosoma and forelegs (WB_plus), mites without both the gnathosoma and forelegs (WB_minus), gnathosoma or forelegs. The data are the total number of transcripts detected or upregulated in each group

Chemosensory group	Total number of chemosensory transcripts detected and upregulated				
	Detected in whole body	Upregulated in WB_plus	Upregulated in WB_minus	Upregulated in gnathosoma	Upregulated in forelegs
**Soluble carrier proteins**					
Insect OBPs	5	–	1	2	1
OBP-like	2	1	1	1	1
NPC2	4	1	–	–	1
lipocalins	6	1	2	–	3
**Receptors and membrane bound proteins**					
IGRs	25	2	1	2	–
IRs	1	–	–	–	1
GRs	2	–	–	1	1
SNMPs	5	–	1	–	–
TRPs	21	–	3	2	–
ENaCs	12	–	1	–	3

–No transcript was upregulated

### Silencing of target LOC111245174 transcript

The survival rates after 15 h of soaking in saline or Vd105872-dsRNA solutions 2.5 µg/µl and 4 µg/µl solution of dsRNA and a control template were: 77% vs. 65%, 63% and 69% respectively, However, the GR gene transcript (LOC111245174) was not silenced in mites soaked in either 2.0, 2.5–4 µg/µl dsRNA solution when compared to non-silenced control mites (One-way Anova: F_(2,16_) = 0.16, *p* = 0.85, Supplementary Fig. S2).

## Discussion

Through transcriptomic and proteomic analyses, our results revealed that *Varroa*’s gnathosoma expresses proteins from the same groups of putative chemosensory transcripts that were previously reported in the forelegs (Eliash et al. 2019) including lipocalin (Fig. 2). This suggests the existence of two functional chemosensory systems in *Varroa* that share some common molecular features. Yet, both organs differ in the expression profiles of some putative lipid carrier proteins (OBP-like, NPC2 and lipocalins), membrane-bound receptors and associated proteins (IGRs, IR, TRPs and ENaCs) (Fig. 2). This further suggests that the two organs may not necessarily perform the same sensory functions since we previously demonstrated that the gnathosoma sensilla have a minor chemosensory function in terms of host recognition when compared to those of the forelegs (Nganso et al. 2020).

The lack of the CSP lipid carrier and the OR groups in the whole body of the mite strongly supports previous studies that reported the absence of these chemosensory groups in *Varroa* (Eliash et al. 2017, 2019; Iovinella et al. 2018). Although the CSP group has been reported in the genomes of all arthropods (Gulia-Nuss et al. 2016; Eyun et al. 2017), including two chelicerate species: the Baja California bark scorpion, *Centruroides exilicauda*, and the western black widow spider, *Lactrodectus hesperus* (Eyun et al. 2017) and one Acari, *Ixodes scapularis* (Gulia-Nuss et al. 2016; Eyun et al. 2017), their function in Acari remains inconclusive. Further, our findings confirm once again the complete absence of OR genes in Chelicerata examined to date (Brand et al. 2018; Eyun et al. 2017; Vizueta et al. 2018). Taken together, our results suggest that the molecular features of these chemosensory organs resemble mostly those of their close chelicerate relatives and in some ways those of their distantly related Crustacea and Myriapoda relatives, as demonstrated before (Vizueta et al. 2018).

### Chemosensory transcripts are expressed in all body parts

In this study, it was interesting to note that all 83 chemosensory transcripts are also expressed in the whole body and the whole body devoid of the two chemosensory appendages (Fig. 2). In particular, seven of these 83 transcripts were highly expressed in the whole body devoid of the two chemosensory appendages compared to the gnathosoma (Fig. 2). They include one insect OBP (LOC111243821) and OBP-like (LOC111247024), two lipocalins (LOC111249022, LOC111250379), one TRP (LOC111251668, LOC111243271) and ENaC (LOC111253877) transcripts. Indeed, it has been reported that some of these chemosensory genes such as OBPs and NPC2s (Pelosi et al. 2014, 2018; Bhowmick et al. 2020), lipocalins (Zhu et al. 2021a), GRs and IRs (Ngoc et al. 2016; Vizueta et al. 2018), TRPs (Kozma et al. 2018), ENaCs (Ben-Shahar 2011; Ngoc et al. 2016) are also expressed in other parts of the body. This suggests that chemosensory functions of these transcripts are likely to extend to other body parts or their functions may extend beyond chemoreception of the external cues in some arthropod groups including mites and ticks, similar to findings in vertebrates (Berg and Kaunitz 2016). Future annotation studies via techniques such as RNA interference (RNAi) or the bacterial type II Clustered Regularly Interspaced Short Palindromic Repeats and associated protein 9 (CRISPR-Cas9) system (Nganso et al. 2022) will be needed to enhance our understanding of the role of these transcripts in mite’s body function.

### Soluble carrier proteins function in both short- and long-range chemoreception

Of the seven OBP transcripts found in this study, six were originally reported in *Varroa*’s forelegs (Fig. 2). The new OBP transcript detected in this study (LOC111255133), was classified as an insect OBP-related transcript because it contains both the conserved insect-OBP domain (IPR036728) and the GO-term of “odorant binding” (GO-0005549). It is worth mentioning that our previous exclusion assay’s study demonstrated that two OBP transcripts previously reported in *Varroa* (Eliash et al. 2019) were foreleg-specific as their expression levels dropped significantly following the removal of the forelegs (Nganso et al. 2021). In this study, we detected only one out of these two transcripts (LOC111247116) in gnathosoma, but neither of the corresponding protein products were found in the gnathosoma proteome of this study or in the foreleg proteome from previous studies (Eliash et al. 2019’s study). This might indicate a short lifespan or low abundance of the protein product of this transcript (Nganso et al. 2022). Moreover, the sensitivity of the methods used in the previous and this study could also explain the lack of detection of the protein product of this transcript.

Interestingly, a specific NPC2 transcript (LOC111247660) was significantly upregulated in the forelegs when compared to the rear legs and the gnathosoma relative to the whole body without chemosensory organs (Fig. 2). This result confirmed our previous exclusion study that demonstrated that the expression level of this transcript dropped significantly in the absence of the forelegs (Nganso et al. 2021). Knockdown of this highly expressed and foreleg-specific transcript via RNA interference suggests that it might be involved in the detection of short-range cues because *Varroa*’s feeding and reproduction were significantly reduced following its silencing (Nganso et al. 2021). Similarly, a second NPC2 transcript (LOC111247342), whose expression was shown to be similar in both the foreleg and the gnathosoma via the exclusion assay (Nganso et al. 2021), appears to be expressed at low levels in both chemosensory appendages (Fig. 2). Contrary to the first annotated NPC2 transcript, silencing this transcript affected *Varroa*-host reaching ability suggesting its involvement in the detection of long-range cues (Mani et al. 2022). Given these results, it seems that both appendages are equipped with lipid binding proteins enabling them to solubilize different odorant molecules and carry the latter to the chemoreceptors in the chemosensory sensilla.

Six out of the nine lipocalins reported in the genome of *Varroa* mite (Zhu et al. 2021a) were found in this study (Fig. 2). Of the six sequences of lipocalins detected, it was interesting to note that three (LOC111247224, LOC111251803 and LOC111251576) were highly expressed in the mite forelegs compared to the rear legs, and none in the gnathosoma. The fact that the corresponding protein products of none of these upregulated lipocalin transcripts were not detected in the gnathosoma’s proteome may be due to the factors highlighted above and may suggest that the CRISPR-Cas9 system might be used in future for their functional annotation (Nganso et al. 2022). Overall, our results indicate that in the absence of CSP, these lipocalin transcripts could represent another group of semiochemical carriers in *Varroa* in particular as well as in other chelicerates, crustaceans and hexapods in which they were found to be highly expressed in chemosensory structures earlier by Zhu et al. (2021a).

### Taste and olfactory chemoreceptor’s potential role in short range chemoperception

As opposed to the somewhat general expression of the soluble carrier proteins, transcripts of the membrane-bound receptors of the IGR and GR families were upregulated in the gnathosoma more than in any other body parts (Fig. 2). Proteins of these groups are known to be involved in olfaction (smell) and gustation (taste) in chelicerates in the absence of OR group (Vizueta et al. 2018; Su et al. 2021). The gnathosoma-specific expression of these transcripts could be adaptive as the mite primarily uses its gnathosoma to sense and taste its suitable host stage to ensure its feeding and reproduction, whereas the forelegs are primarily raised above the surface in a similar manner to the antennae of other arthropods for volatile detection. In fact, the two IGR transcripts (LOC111247324 and LOC111248674) were significantly (or close to significant) upregulated in the gnathosoma, while none were upregulated in the foreleg (Fig. 2). Both of these upregulated transcripts contained the two essential characteristic domains of the IGR superfamily (IPR019594 and IPR001320) and were also present in the proteome of the gnathosoma, suggesting that their proteins are abundant. Moreover, one GR transcript (LOC111245174, referred to as *Vd105872* in Eliash et al. 2017) was significantly upregulated in the gnathosoma and the forelegs, suggesting that it is probably involved in both gustation and olfaction. The fact that it was not found in the proteome of neither gnathosoma (this study) nor in the foreleg in Eliash et al. 2019 may as in previous cases suggest low abundance and/or short life of its protein. Intriguingly, our attempts to silence the expression of this gene by RNAi failed and could be due to the factors mentioned above. Further, not all genes can be silenced by RNAi as gene silencing is a “tricky” operation and may be impacted by the interaction between the siRNA and the other RNAi pathways (Ghildiyal and Zamore 2009; Yang et al. 2020; Nganso et al. 2022). Perhaps, functional analyses using other approaches such as CRISPR-Cas9 (the bacterial type II Clustered Regularly Interspaced Short Palindromic Repeats and associated protein 9 system) and/or the Xenopus oocyte expression system could elucidate the function of this gene in *Varroa*-honey bee chemosensing The other GR (GR2) transcript (LOC111254617, referred to as *Vd7144* in Eliash et al. 2017) was not upregulated in the foreleg or gnathosoma, and its product was absent from the proteome of the gnathosoma and in the former foreleg’s proteome (Eliash et al. 2019). It is worth indicating that this GR2 transcript is apparently an isoform of an additional GR transcript found previously in *Varroa*’ forelegs (Eliash et al. 2017, 2019; former name Trinity_DN29661_c0_g1) and named there as GR3. In *Varroa* forelegs, only one potential IR transcript (LOC111251297, referred to as *Vd17150* in Eliash et al. 2017), which is an IR25a-like homologue and contains the two essential characteristic domains of the IGR superfamily was significantly upregulated (Fig. 2). But the product of this transcript was not found in the proteome of the gnathosoma (in this study) and that of the foreleg (Eliash et al. 2019). We also found that this IR25a transcript is an isoform of an additional IR (IR1) transcript (referred to as Trinity_DN26056_c0_g1_i2 and *Vd19098* in Eliash et al. 2019 and 2017; respectively) found previously in *Varroa* forelegs (Eliash et al. 2017, 2019). Recently, we showed that the IR25a (LOC111251297) and GR1 (LOC111245174) transcripts are co-expressed with transcripts of some lipid carrier proteins in the putative gene networks we built, further supporting their role in *Varroa*’s chemoreception (Mani et al. 2022).

Among the SNMP chemosensory associated proteins, which contain the conserved domain of the CD36 receptor family, our study did not reveal any significantly upregulated transcripts in either the foreleg or gnathosoma (Fig. 2). This suggests that this group is unlikely to play a role in chemosensation in *Varroa*. It is worth mentioning that members of the CD36-SNMP group have been reported in other arthropods including hexapods, myriapods and crustaceans studied to date (Vizueta et al. 2018; Su et al. 2021), and recently their role in sex pheromone detection in *Bombyx mori* was demonstrated (Zhang et al. 2018). Regarding the specialized ion channel receptor families of TRP and ENaC, two TRP transcripts (LOC111243805, LOC111248665) were significantly upregulated in the gnathosoma but no ENaC transcripts were upregulated in this organ (Fig. 2). On the other hand, in the mite’s forelegs, none of the potential TRPs were upregulated while three ENaCs (LOC111254848, LOC111244963 and LOC111251329) were significantly upregulated. This suggests that transcripts of TRP and ENaCs, which are upregulated in the chemosensory appendages, could play a role in *Varroa*-honey bee interaction. Co-expression networks presented in previous study also pointed towards the involvement of TRP1 in this process (Mani et al. 2022). Orthologs of ENaCs and TRPs were reported in other arthropods including mites and ticks (Ngoc et al. 2016; Kozma et al. 2020), but their role in chemoreception has not yet been extensively elucidated. However, the role of TRP in reception of plant-derived volatiles was recently demonstrated both in Chelicerates, specifically in *V. destructor* (Peng et al. 2015), and insects (Tian et al. 2022). The latter manuscripts further suggested this receptor as a potential target in development of new methods for Arthropod pest management.

In conclusion, comparative transcriptomic and proteomic analyses revealed the presence of the putative chemosensory related transcripts belonging to several groups based on conserved domain search in the whole body, gnathosoma, and body devoid of the two chemosensory organs of the honey bee parasitic *Varroa* mite. To the best of our knowledge, this is the first study that compares the expression of transcripts from several chemosensory-related groups between these body parts of a mite. Through this study, we confirm that *Varroa*’s gnathosoma possesses chemosensory abilities given the expression of families of chemosensory genes that were previously reported in the mite’s forelegs (Eliash et al. 2017, 2019) as well as additional ones. The exact site of chemosensory organs in gnathosoma is still not clear, though some sensilla on the mite’s palpi were found before (Liu and Peng 1990). Given the differences in the expression profiles of some putative chemosensory genes between the forelegs and the gnathosoma, our results suggest that these two chemosensory systems apparently differ in characteristics of compounds that are detected. These differences in detection abilities will require further studies, as well as the function of the putative chemosensory transcripts found in the body deprived forelegs and gnathosoma. Could there be additional chemosensory organs not yet revealed? On the other hand, could these transcripts have other roles such as in internal body communication? Our results emphasize the complexity of detection and annotation of chemosensory-related proteins that involves the combination of both behavioral and molecular approaches. Ideally, the combination of the molecular manipulations such as gene-knockdown (RNAi) or CRISPR-cas9, in addition to the recently improved tool of protein fold prediction (Jumper et al. 2021), will help to confirm the role of suspected proteins, and more importantly, the detection of new ones.

## Electronic supplementary material

Below is the link to the electronic supplementary material.

Supplementary Table S1 Figure S1 and Figure S2

Supplementary Table S2

Supplementary Table S3

## Data Availability

The raw read sequences from the three RNA samples from *Varroa* mite were uploaded to the National Centre for Biotechnology Information (NCBI) on accession No° PRJNA872955. Proteomic data are available via ProteomeXchange with identifier PXD053065.
