# Supplementary material for: Chemosensory function of Varroa gnathosoma: transcriptomic and proteomic analyses
Source: Exp Appl Acarol. 2024 Oct 23;93(4):701–19. doi: 10.1007/s10493-024-00952-1 (PMC11534843; doi:10.1007/s10493-024-00952-1)
Supplement: Supplementary file 1 — Supplementary Table S1 Figure S1 and Figure S2 [file 10493_2024_952_MOESM2_ESM.docx]

**Table S1.** List of pair of primers for dsRNA synthesis of target gene transcript and RT-qPCR evaluation of gene silencing. Bold letters indicate sequences of T7 promoter.

| **Transcript category** | **Locus ID** | **Transcript name *#*** | **Primers’ sequences** | **Annealing temperature (Tm °C)** | **Product length (bp)** |  | **Source** |
| --- | --- | --- | --- | --- | --- | --- | --- |
| GR | LOC111245174 | TRINITY_DN23243_c0_g1_i2 | Fwd 5'-**TAATACGACTCACTATAGGGAGACCAC** GTCACTCGAGCTCTCTACGC-3'  Rev 5'- **TAATACGACTCACTATAGGGAGACCAC** AGCTCGTCAAGTGTGTCCAG-3' | 60 | 158 | dsRNA synthesis | <https://www.ncbi.nlm.nih.gov/tools/primer-blast/> |
|  |  |  | Fwd 5'-AAAGAGCGCGACAATACCCA-3'  Rev 5'-ATGTTGTGACCAGATGCCGT-3' | 60 | 134 | RT-qPCR |  |
|  |  |  |  |  |  |  |  |
|  |  | Fwd 5'-**TAATACGACTCACTATAGGGAGACCAC** AGTGTGTAGGGACTCGGTAA-3'  Rev 5'- **TAATACGACTCACTATAGGGAGACCAC** GGAATCCGGAAGTAGCGATAAG-3' | 62 | 230 | dsRNA synthesis | <https://eu.idtdna.com/PrimerQuest/Home/Results> |  |
|  |  |  | Fwd 5'-GTTCAAAGAGCGCGACAATAC-3'  Rev 5'-GACCGTTCCGACTAACGTAAA -3' | 62 | 175 | RT-qPCR |  |

***#***Transcript nomenclature according to Eliash et al. 2019


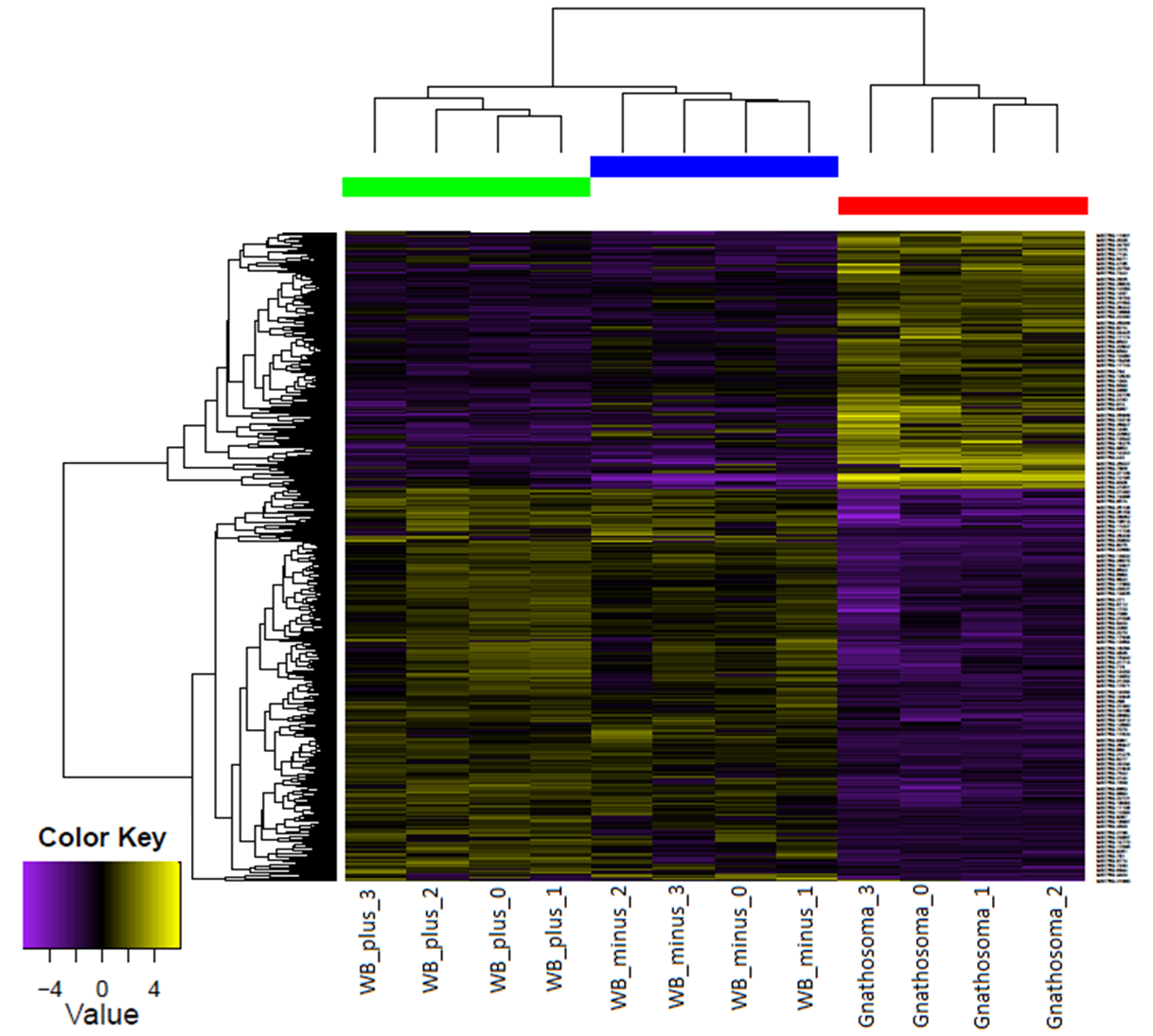


**Figure S1:** Heat map of the 2067 differentially expressed genes for the three RNAs groups: gnathosoma only (gnathosoma), mites without gnathosoma and forelegs (WB_minus), and mites with both the gnathosoma and forelegs (WB_plus). Log2 of the normalized reads are displayed as the color scale with downregulated genes in purple and upregulated genes in yellow.

a

b

**Figure S2**. The analysis of the expression of GR gene transcript after 15 hrs of mite silencing with: **a.** first set of primers and **b**, the second set of primers. The numbers above each bar indicate the sample size per treatment group: Control (saline), dsGR 2.5 µg/µl & 4 µg/µl in saline). Data were normalized using 18S rRNA and expressed as the mean ± SE. The relative expression level was calculated using the **2^−△△Ct^** method. No significant difference was found in the expression levels target transcript between the treatment groups (one-way ANOVA (p>0.05).
